# Oxidative Stress, Testicular Inflammatory Pathways, and Male Reproduction

**DOI:** 10.3390/ijms221810043

**Published:** 2021-09-17

**Authors:** Sulagna Dutta, Pallav Sengupta, Petr Slama, Shubhadeep Roychoudhury

**Affiliations:** 1 Faculty of Dentistry, MAHSA University, Shah Alam 42610, Malaysia; sulagna_dutta11@yahoo.com; 2 Faculty of Medicine, Bioscience and Nursing, MAHSA University, Shah Alam 42610, Malaysia; pallav_cu@yahoo.com; 3 Department of Animal Morphology, Physiology and Genetics, Faculty of AgriSciences, Mendel University in Brno, Zemedelska 1, 613 00 Brno, Czech Republic; petr.slama@mendelu.cz; 4 Department of Life Science and Bioinformatics, Assam University, Silchar 788011, India

**Keywords:** cytokines, inflammation, male infertility, oxidative stress, reactive oxygen species

## Abstract

Inflammation is among the core causatives of male infertility. Despite male infertility being a serious global issue, “bits and pieces” of its complex etiopathology still remain missing. During inflammation, levels of proinflammatory mediators in the male reproductive tract are greater than usual. According to epidemiological research, in numerous cases of male infertility, patients suffer from acute or chronic inflammation of the genitourinary tract which typically occurs without symptoms. Inflammatory responses in the male genital system are inextricably linked to oxidative stress (OS). OS is detrimental to male fertility parameters as it causes oxidative damage to reproductive cells and intracellular components. Multifarious male infertility causative factors pave the way for impairing male reproductive functions via the common mechanisms of OS and inflammation, both of which are interlinked pathophysiological processes, and the occurrence of any one of them induces the other. Both processes may be simultaneously found in the pathogenesis of male infertility. Thus, the present article aims to explain the role of inflammation and OS in male infertility in detail, as well as to show the mechanistic pathways that link causative factors of male reproductive tract inflammation, OS induction, and oxidant-sensitive cellular cascades leading to male infertility.

## 1. Introduction

**Figure 1:**
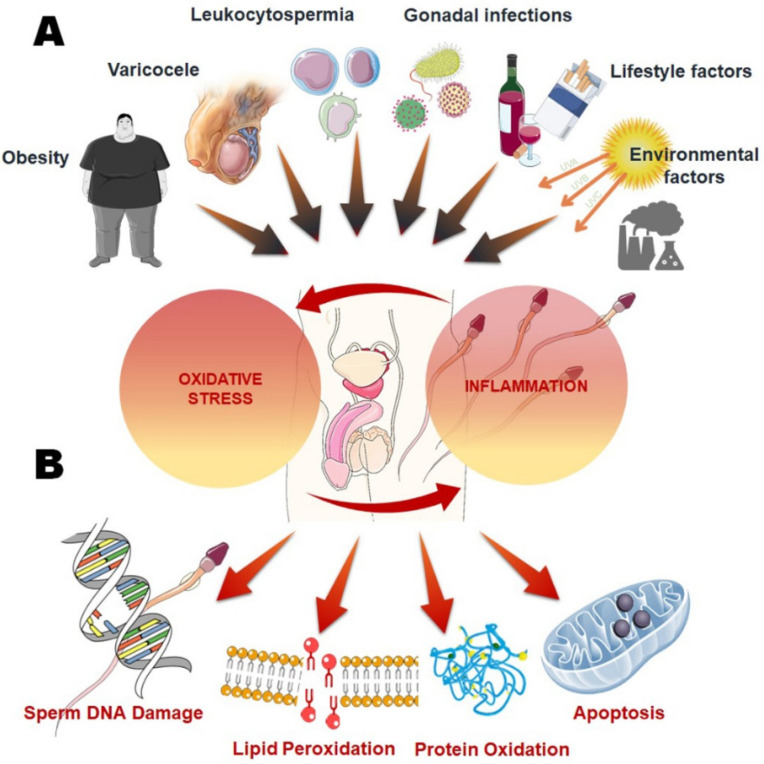
Inflammation and oxidative stress (OS) as core mechanisms linking common causative factors with male infertility. (**A**) Exogenous and endogenous factors impacting on male reproductive system via induction of OS and inflammation; (**B**) Effects of various causative factors mediated male reproductive disruptions mainly via oxidative damage and induction of apoptosis.

Male infertility is a condition that only gained clinical and research attention in the recent decades. The global concern encompassing male factor infertility surged to a higher level when reports pertaining to a declining trend in male fertility parameters were showcased from different geographical areas [1,2,3,4,5]. Even with great advents in male infertility research, the multifarious underlying mechanisms responsible for this condition await complete understanding. In this aspect, it is essential to dig out the common mechanisms that are at the nodal point among all other causatives, linking factors to the ultimate pathological outcome. Numerous factors can impair male fertility, including physiological factors (age, weight, body composition, etc.), pathological (metabolic syndrome, infection, inflammation, etc.), psychological (anxiety, trauma, stress, etc.), lifestyle (physical activities, smoking, alcohol consumption, drug abuse, etc.), as well as environmental (exposure to toxins, heavy metals, radiations, etc.) [6,7,8,9]. All the factors mentioned here and beyond can induce oxidative stress (OS), which represents a common mechanism in the prognosis of male infertility and offers diagnostic answer to most cases of idiopathic male infertility [10]. Inflammation of the male reproductive tract intricately links OS and establishes vicious pathways that structurally and functionally impair male reproductive tissues [11] (Figure 1). The result is an exaggeration of cellular damage and chronic diseases that include disruption of male reproductive tissues affecting male fertility [12,13].

Male factor infertility is principal contributor in almost half of overall worldwide infertility cases, and 25% of male infertility cases remain idiopathic [14]. OS-associated mechanisms are thought to be responsible for disruption of male fertility parameters in approximately 40% to 50% of instances [15,16]. When OS is induced, the endogenous testicular antioxidant defense mechanisms are inadequate to neutralise the damaging effects of excess reactive oxygen species (ROS) [16]. ROS (such as the superoxide anion, O_2_•−, hydroxyl radical, HO•, and hydrogen peroxide, H_2_O_2_) are extensively reactive metabolism-derived oxidising molecules [15]. At normal, physiological levels, ROS are crucial for male reproductive functions such as spermatogenesis, to sustain sperm viability, to mediate maturation, hyperactivation and capacitation, sperm motility as well as in acrosome reaction (AR) [16]. Maturation of spermatozoa occurs in the epididymis; there occurs a series of events including changes in the sperm plasma membrane, rearrangement of the membrane proteins, alterations in the enzymes, and nuclear remodeling [17]. Seminal ROS-regulated signalling pathways are essential for these maturation events [18,19]. Since smaller-sized protamines replace the histones as nuclear proteins in mammalian spermatozoa, it is possible to highly condense the DNA in an array type structure instead of supercoiled solenoids [20]. The cysteine residues of the protamines have inter- and intramolecular disulfide bonds between them to impart chromatin stability [21]. ROS facilitates the formation of disulfide bonds, rendering chromatin stability and preventing damage to the chromosomal DNA. ROS, especially peroxides, also play a role in the mitochondrial capsule formation, formed by a network of proteins with several disulfide bonds preventing proteolytic degradation of mitochondria [17,22]. ROS also play a critical role in seminal coagulation and liquefaction processes required for the transport of sperm to the female reproductive tract [23]. ROS are important for sperm capacitation and hyperactivation. These processes are mediated by induction of Ca^2+^ and HCO_3_^−^ influx, inactivation of plasma membrane Ca^2+^-ATPase (PMCA), and cytosol alkalisation. Calcium ions and ROS mediate sperm acrosome activation and upregulate cyclic AMP, which activates intracellular protein kinase A and the subsequent signalling pathway needed for sperm hyperactivation and capacitation [19]. Throughout the duration of spermatozoa capacitation, ROS prevent the deactivation of phospholipase A2 (PLA2) via inhibition of protein tyrosine phosphatase activity. Thus, the activated PLA2 potentially cleaves the secondary fatty acid from the membrane phospholipid triglycerols and enhances the plasma membrane fluidity required for sperm–oocyte fusion [24].

However, supraphysiological concentration of ROS affects typical sperm parameters such as sperm motility, capacitation, acrosome reaction, and oocyte penetration, all of which are crucial for conception [15,16]. During spermatogenesis, haploid spermatozoa are produced in the seminiferous tubules. Sertoli cells containing antioxidant enzymes protect the developing germ cells from testicular OS [25,26]. Since they are no longer shielded by the Sertoli cell defense mechanism, spermatozoa become vulnerable to oxidative damage as they are pushed out of the germinal epithelium [15,27]. Excess ROS in cells can affect intracellular components, nucleic acid, lipids, and proteins, resulting in cellular injury [15,28,29].

Reproductive tract inflammation activates and attracts immune cells at the site of inflammation that cause burst of excess reactive substances, exacerbating OS [30]. On the other hand, certain ROS and/or reactive nitrogen species (RNS) can activate intracellular signalling cascade that promotes the activation of proinflammatory genes [31,32]. Inflammation and OS are thereby pathophysiological phenomena that are inextricably intertwined. The unique inflammatory responses of the male reproductive tissue, the inflammatory stimuli, the elicited cellular pathways as well as the inflammation–OS loop resulting in male infertility/subfertility have not been comprehensively explained. Thus, the present article aims to comprehensively explain the role of inflammation and OS in male infertility, as well as to illustrate the mechanistic pathways that associate causative factors of male reproductive tract inflammation, induction of OS followed by the oxidant-sensitive cellular cascades leading to male infertility.

## 2. Causes of Inflammation in Male Reproductive Tract

Inflammation in the male reproductive tract can be caused and facilitated by multifarious factors (Figure 1). The most common underlying causatives of male reproductive tract inflammation are discussed here. Ejaculatory duct obstruction is a prevalent cause of male infertility, and infections have been found in at least 20% to 50% of these individuals [33]. Epididymitis is an inflammation of the epididymis, which connects the testes with the vas deferens in the male reproductive system. Further problems from epididymis inflammation include scrotal enlargement, discomfort, penile discharge, and blood in the urine. Other major sources of inflammation are the sexually transmitted infections such as gonorrhoea, *Chlamydia*, and *Escherichia coli*, which constitute the most common causes of epididymitis in older men, although other bacteria such as *Mycobacteria* and *Ureaplasma* are also accountable. Next urethritis is a mentionable source of inflammation. It refers to bladder or urethral infection that spreads to the epididymis. Various viral infections can also cause inflammation and thereby curb male fertility [34]. Moreover, inflammation may also be mediated by testicular torsion, which is a frequent reproductive disorder owing to a supporting tissue defect that causes the testes to twist inside the scrotum, resulting in severe edema. Torsion induces testicular injury by disrupting the blood arteries that supply the testes. A varicocele is an expansion of the internal spermatic veins, which drains blood from the testicle to the abdomen (back to the heart). When the one-way valves in the spermatic veins become impaired, an aberrant backflow of blood into the scrotum occurs, providing an unfavorable environment for sperm growth [35].

When one or more of these discussed processes occur in the male genital tract, unrestricted immune responses are elicited. This form of inflammation causes collateral damage that builds up over time, often asymptomatically for years, and eventually leads to tissue damage, thereby compromising male reproductive functions [36]. While the goal of the procedure is to prevent damage and restore function, the reaction itself can be harmful as inflammation has the unintended effect of releasing enzymes and toxic chemicals from phagocytic tissues, causing damage to cells and tissues.

### 2.1. Endogenous Causes of Inflammation

#### 2.1.1. Obesity

There is evidence that obesity causes systemic inflammation that progresses to a chronic inflammation dependent on T helper 1 (TH-1) T lymphocytes [37,38]. The progression of the inflammatory process gradually impairs the functions of critical organs, with no exception for the male reproductive tissues, which include the testicles, epididymis, and male accessory glands [37,39]. The position of the testicles, which is slightly outside the abdominal cavity, as well as their anatomical peculiarities, make them more susceptible to infection and inflammation [37]. Several pro-inflammatory mediators, including the cytokines [40], interact with the complex reproductive regulation of the HPG axis, resulting in alterations in the function of the testicles [41]. It most often impairs steroidogenesis or spermatogenesis that is responsible for hypogonadotropic hypogonadism as well as decreased semen parameters in men. As a result, inflammation may result in the excessive production of ROS and the promotion of testicular OS [27,42].

Because of the ability to directly affect testicular cells and disrupt spermatogenesis, ROS can serve as an independent indication of male factor infertility/subfertility. Because of their highly reactive qualities, when the level of seminal ROS surpasses physiological values, they generate a state of OS, which causes considerable disruption of the functions and morphology of the cells. Multiple studies have indicated that obesity and its associated systemic diseases are associated with elevated testicular OS [42,43,44]. Most likely, the greater metabolic rates required to sustain normal biological activities, as well as elevated stress levels in the local testicular environment, are the causes of this correlation [45]. Both these factors are known to generate ROS in the body. Sperm DNA damage, deformation, and compromised plasma membrane integrity have been observed [46,47]. Through damage to mitochondrial genomes, ROS influence sperm motility, resulting in altered sperm mitochondrial activities and reduced energy production in the spermatozoa. Furthermore, OS is a component of the pathophysiological process of erectile dysfunction, which may explain why obese persons have a higher incidence of the condition.

#### 2.1.2. Varicocele

Varicocele is a condition in which the pampiniform venous plexus inside the spermatic cord dilates and twists abnormally [48]. Varicocele is diagnosed in 15% to 20% of males with reproductive issues worldwide [49], resulting in higher seminal ROS levels and reduced overall antioxidant capacity [50]. Varicocele causes spermatic vein ischaemia, which leads to higher amounts of inflammatory cytokines and nitric oxide (NO) [51]. The physiological functions of cells inside the testes are maintained by moderate amounts of particular cytokines. Interleukin (IL)1 affects the functioning of testicular cells such as Sertoli and Leydig cells in particular [52]. Furthermore, certain cytokines, such as IL37 and IL18, are increased in varicocele patients’ seminal plasma [53]. These levels cause inflammatory response activation, leucocyte recruitment, and the formation of ROS, all of which are harmful to normal testicular function [54]. As a result, ROS can wreak havoc on the blood–testis barrier, sperm plasma membrane, and DNA integrity [10]. Additionally, in varicocele testes, endothelial NO synthase (eNOS) is increased to enhance blood flow into testicular tissues and compensate for hypoxia produced by venous stagnation [48]. High NO concentrations in this scenario can be harmful because NO can react with superoxide free radicals to generate extreme RNS such as peroxynitrite and peroxynitrous acid, which can cause infertility [55]. Leptin receptors, glial cell line derived neurotrophic factor receptor 1 (GDNF1), and voltage-dependent calcium channels are all probable causes of OS in varicocele patients [56].

Laboratory indications of inflammatory status include mean platelet volume (MPV), seminal epithelial neutrophil activating peptide-78 (ENA-78), and seminal IL1B. When compared with fertile men, these indicators were considerably greater in subfertile patients with varicoceles [57,58]. In subfertile males with varicoceles, spermatozoa motility is reduced in response to elevated ENA-78 [58], and the increased MPV tends to diminish after varicocelectomy [57]. For varicocele-related infertility, neutrophil products could be employed as diagnostic indicators and treatment targets [58]. In rat testes, an IL-1b antagonist, anakinra, inhibited induced OS and, as a result, histopathological damage in the tunica albuginea, germ cells, seminiferous tubules, and interstitial tissue [59].

#### 2.1.3. Leukocytospermia

Leukocytes are the primary source of ROS in sperm, producing up to 1000 times the amount of ROS produced by normal spermatozoa [60]. Globally, approximately 10–20% of infertile men have high seminal leucocyte concentrations, which can be caused by infections or inflammatory responses, among other things [61]. Particles of foreign material and cytokines are destroyed by polymorphonuclear granulocytes (which account for 50–60% of all leucocytes in the ejaculate) and macrophages (20–30% of WBCs that remove foreign material and produce cytokines) [62]. Macrophages are found throughout the tissues of the body. When activated, macrophages secrete proteases, neutrophil chemotactic factors, and ROS, which signal to the endothelial cells lining the blood vessels to display certain molecules on the inner walls of the blood vessels, resulting in an increase in inflammation [63]. WBCs produce enormous levels of ROS to combat infections and destroy pathogens. This is accomplished by boosting the activity of G6PDH, which results in the synthesis of significant amounts of NADPH. NADPH oxidase also uses an electron from NADPH to convert oxygen into a superoxide anion, which is toxic to cells. As a result, an excessive amount of OS will activate the chemokines CXCL5, CXCL8, IL6, and IL8 [64,65]. A consequent imbalance between antioxidants and seminal ROS levels arises, leading to OS-induced sterility in the female reproductive system [66]. High quantities of seminal leucocytes impair sperm concentration and motility, as well as causing aberrant sperm morphology, in male infertility [67,68]. It is possible that the early detection of novel biomarkers such as TLR2, TLR4, COX2, and Nrf2 will aid in the development of more effective therapeutic approaches for the treatment of OS-induced infertility [69].

### 2.2. Exogenous Causes of Inflammation

#### 2.2.1. Sexually Transmitted Diseases

Sexually transmitted diseases (STDs) affect the hormonal axis, spermatogenesis, or the transport of sperm through the urinary system. Male infertility is mostly caused by STDs; a high titre of seropositive *Chlamydia* antibodies, which is a marker of severe infection, has been found to be associated with male infertility [70]. There was a significant increase in the number of *Chlamydia*-, *Mycoplasma hominis*-, and *Ureaplasma urealyticum*-positive PCR results in infertile men as compared with fertile men, demonstrating that there is a strong link between STD infection and infertility [71]. When spermatozoa were incubated with lipopolysaccharides isolated from *Chlamydia*, the results showed that the spermatozoa died completely and that their motility had decreased [72]. Male infertility has been linked to genital *Mycoplasma* and *Ureaplasma*, which have been shown to cause decreased sperm motility and membrane changes in addition to DNA damage [73]. It has been reported that semen samples from infertile men contain *Neisseria gonorrhoeae* DNA, and *Trichomonas vaginalis*, which has been shown to decrease sperm motility [74]. Men are also susceptible to infection with the Epstein–Barr virus, cytomegalovirus, herpes, papilloma virus, hepatitis, and the human immunodeficiency virus (HIV). However, the association of unknown origin due to STDs has not been extensively studied to establish a strong correlation between individualised STD infection and male infertility.

#### 2.2.2. Prostatitis

Prostatitis continues to be the most thoroughly researched inflammatory disease in the male genital system. In the case of acute and chronic bacterial prostatitis, pathogens can affect sperm directly (depending on the pathogen strain) or indirectly by the activation of cytokines such as IL6, IL8, or tumour necrosis factor-α (TNF-α) [75]. Increased cytokine levels lead to OS, which affects not only the spermatozoa [40], but also has the potential to trigger a systemic reaction by lowering testosterone hormone levels. When the infection extends to the testis, elevated IL6, IL8, and TNF-α levels may further affect sperm transit during ejaculation [76,77]. Increased OS frequently has a direct effect on sperm DNA, reducing the paternal genetic contribution to the embryo [78]. Infection and inflammation of the prostate excite leukocytes, which can produce ROS. As a result, infections must be treated immediately, as a high level of ROS may harm up to 35% of men seeking infertility therapy [79]. This is mostly due to the fact that untreated prostatitis can result in oligozoospermia, azoospermia, or asthenozoospermia [80]. Chronic sickness, which is more difficult to treat than acute disease, can also be caused by untreated/undertreated infections. Four specific antibiotics were offered as therapy options for bacterial prostatitis: fluoroquinolones, tetracyclines, macrolides, and trimethoprim (alone or in combination with sulfamethoxazole) [81]. Antibiotic therapy remains the cornerstone of bacterial prostatitis treatment, and multiple studies have demonstrated that antibiotics can significantly enhance sperm parameters and pregnancy rates. Nonetheless, antibiotic treatment must be carried out cautiously and following appropriate drug resistance testing, as current research has focused on multidrug resistance [82]. Combination therapy is often indicated for individuals whose first-line therapy is ineffective [83]. While the majority of combinations are ineffective or additive, ciprofloxacin and rifampin appear to be effective against *Staphylococcus aureus* [84], and fluoroquinolones chelate with metal cations such as aluminium, magnesium, calcium, iron, and zinc. This pharmacological interaction dramatically lowers the serum drug concentrations available for tissue penetration [84]. Prostate epithelial cells can create three times the amount of zinc found in most other mammalian cells [85]. Despite the preceding description, zinc accumulation in the prostate may hinder fluoroquinolones from obtaining their maximum efficiency.

#### 2.2.3. Urethritis

Infection or inflammation of the urethra may result in infertility due to the direct impact of colonised bacteria or other pathogens on spermatozoa when they travel through the urethra during ejaculation. Tissue scarring as a result of infection may restrict sperm deposition in the female reproductive tract by affecting the semen volume and/or the sperm count [86]. Urethritis is a genitourinary disease that is frequently observed among men. A number of pathogens, including Mycoplasma genitalium (MG), Neisseria gonorrhoeae (GC), Trichomonas vaginalis (TV), Chlamydia trachomatis (CT), Ureaplasma urealyticum (UU), herpes simplex virus (HSV), and adenovirus, have been linked to this inflammatory condition [87]. The coagulase-negative staphylococcus Staphylococcus saprophyticus causes frequent urinary tract infection in women. It has, however, been proven to induce urethral infection in males, but its significance in this regard is unclear [88,89]. It has been linked to a variety of complications, including acute epididymitis, orchitis, and prostatitis, among others.

#### 2.2.4. Viral Infections

##### HIV

HIV lowers sperm motility, vitality, and forward advancement when the CD4 cell count is fewer than 350 cells per microliter [34], which occurs when the virus is present. Asymptomatic HIV-seropositive individuals do not have diminished fertility [34] unless they are infected with the virus. People with symptomatic HIV infections have been shown to have increased recruitment of monocytes, macrophages, and leukocytes in their sperm [34]. Despite the fact that current highly active antiretroviral therapy (HAART) regimens are increasing the life expectancy of HIV-infected men, research on how HAART impacts male fertility has revealed that it has a negative impact on the quality of sperm [90]. Since non-nucleoside reverse transcriptase inhibitors are mitochondrially toxic [91,92] and cause disruption to the mitochondrial membrane potential [93], their use is associated with an increase in OS.

##### Hepatitis

It has been demonstrated that the hepatitis B and C viruses are responsible for male infertility. Because of its capacity to pass through the blood–testis barrier, hepatitis B has been shown to worsen parameters of fertility [34,94]. It has been demonstrated that, due to the virus’s capability of crossing across into the testicles from the bloodstream, it can transmit its genome straight into the spermatozoa, resulting in faulty spermiogenesis and reduced fertilisation levels [95]. Phosphatidylserine externalisation was enhanced in spermatozoa from hepatitis B-exposed individuals, and lipid peroxidation (LPO) was measured by the production of MDA in these individuals [96,97]. Men with chronic hepatitis B were shown to have high seminal quantities of the proinflammatory cytokine IL18, which stimulates natural killer cells to release the proinflammatory cytokine IFN-Ɣ [97]. These researchers discovered a positive relationship between the production of inner MDA and the concentration of IL18, showing that the formation of MDA is a result of an inflammatory process. Although there have been some investigations into the relationship between IL18 and leucocyte activation in the male reproductive tract as a result of hepatitis B infection, there has been no direct connection found. Intravenous drug misuse and mother-to-child transmission are the most common routes of infection for hepatitis C [98,99]. The ongoing opiate epidemic in the United States is primarily characterised by the presence of reproductive-age opiate addicts (with an average age of 22.9 years) who contribute considerably to the increase in hepatitis C transmission [98]. It should be noted that, in contrast to hepatitis B, the hepatitis C virus does not pass through the blood–testis barrier and so cannot induce direct oxidative damage to spermatozoa [34]. When people suffer from chronic hepatitis C, their TNF-α and NO levels rise throughout their bodies [100]. A chronic inflammatory response, lymphocyte activation, and polymorphonuclear leukocyte activation are observed as a result [101]. The production of ROS by polymorphonuclear leucocytes via NOX2 results in a decrease in the mitochondrial membrane potential in spermatozoa, which results in the further propagation of ROS and OS [100,101]. Hepatitis C-induced OS causes sperm motility to diminish, but does not influence ejaculatory volume, apoptosis, or DNA damage in the sperm [100,101].

##### Coronaviruses

Researchers have been reporting the possibility of coronaviruses infecting the human reproductive system since the SARS-CoV pandemic in 2002 [102,103,104]. SARS-CoV-2 is thought to operate similarly to SARS-CoV-1 when it comes to disrupting reproductive processes. Men have higher ACE2 receptor expression than women, which might explain why the male reproductive system appears to be more susceptible to SARS-CoV infection [103]. Coronaviruses related to past outbreaks have been linked to orchitis with SARS-CoV being a notable example. This might cause spermatogenesis to be disrupted and germ cells to undergo apoptosis [103]. Immunohistochemistry confirmed traces of IgG deposition in testicular tissues [105]; however, there are controversial reports on the presence of viral genomic components in the testes or semen [106]. Thus, elicitation of testicular innate inflammatory pathways via secondary immune responses plays a key role in the development of virus-mediated testicular damage and the production of OS [107]. Furthermore, similar to SARS-CoV, SARS-CoV-2 is likely to adopt the stress-evasion strategy employing its amino acid sequences that resemble the host’s adrenocorticotropic hormone (ACTH) and, as a result, develop antibodies against the host’s self-ACTH molecules. This method reduces stress and inflammation in the organism by suppressing the stress response in the host, which is typically done by raising cortisol levels [108,109]. As a result, uncontrolled inflammation continues to inflict tissue damage. Membrane lipid peroxidation and sperm DNA fragmentation may occur through intracellular oxidative damage and are detrimental to testicular processes such as spermatogenesis and spermiogenesis [110].

## 3. Oxidative Stress as Cause and Consequence of Inflammation

Inflammation is the body’s reaction to tissue damage and injury. This procedure transports leucocytes and plasma molecules to infection sites and tissues. When there is acute inflammation, three major changes occur: increased blood flow to the afflicted area, increased capillary permeability, which allows bigger serum molecules to enter the tissues, and enhanced leukocyte migration into the tissue. Chronic inflammation results from the inability to remove the infectious pathogen. This is characterised by macrophages, lymphocytes, and other cells being recruited and activated, resulting in a coordinated cytokine response. In contrast to acute inflammation, in which the host’s response results in the irritant’s removal followed by tissue regeneration or repair, chronic inflammation is marked by inflammation and healing occurring simultaneously rather than sequentially. Because chronic inflammation is connected with irritants that destroy tissue architecture, repair is always a component of chronic inflammation. Ingrowth of granulation tissue, which comprises macrophages, fibroblasts, and new blood vessels, is generally used to repair wounds [111].

The clear link between acute or chronic inflammation and the onset of infertility is one of the most pressing concerns in modern medicine. Impaired accessory gland functioning, sperm transport blockage, and spermatogenesis dysregulation may all contribute to worse semen quality during the inflammatory phase [112,113]. Because they are generated by locally activated cells or produced briefly after the stimulus has been triggered, proinflammatory cytokines generally operate locally. Cytokines are also generated physiologically in male gonads and have been implicated in the organ’s proper function [114,115]. They appear to be natural components of seminal plasma in this regard [116]. Testicular macrophages are the major source of cytokines in the male gonad; however, other researchers have reported Leydig and Sertoli cells producing cytokines (IL–1 and IL–6) [117]. When tissue injury occurs, cytokines, notably TNF-α, regulate the migration of leukocytes into tissues. TNF-α is a protein generated predominantly by macrophages and other mononuclear phagocytes that has a role in inflammation and the activation of other leukocytes. TNF-α, for example, stimulates phagocytes’ microbial systems by inducing adhesion molecules and chemokines on the endothelium. TNF-α also causes apoptosis in cells. IL1, similar to TNF-α, is an essential inflammatory cytokine with similar activities [118].

Increased production of ROS is caused by inflammatory injury to the male genital tract. Furthermore, pathogenic bacterial strains that inhabit the reproductive tract may induce ROS or free radical overproduction linked with inflammatory responses [119,120]. Free radicals are a class of extremely reactive molecules that have one or more unpaired electrons and can oxidatively alter biomolecules they encounter. When they react almost instantly with any chemical in their surroundings, they set off a chain reaction that causes cellular damage [121]. The main ROS found in seminal plasma are superoxide, hydroxyl, and hydrogen hydroxide radicals [66].

Excess ROS must be constantly neutralised to preserve normal cell activity. Excess free radicals overpower the antioxidant defences of the male reproductive tract, causing damage to the male reproductive tract [7,122]. Under physiological conditions, excess ROS are scavenged by the endogenous antioxidant system to maintain the seminal redox balance [123]. Endogenous antioxidants can be enzymatic, such as catalase, superoxide dismutase (SOD), thiol peroxidase, and non-enzymatic such as the glutathione [124]. When the levels of seminal plasma ROS overwhelms the endogenous antioxidant capacity, semen parameters may become adversely affected [123,125,126].

Antioxidant genes, which include glutathione peroxidase (GPX), glutathione S-transferase (GST), catalase (CAT), nitric oxide synthase (NOS), keap1 (Kelch-like ECH-associated protein 1) Nrf2 (nuclear factor erythroid 2 related factor 2), and superoxide dismutase (SOD), are crucial for normal spermatogenesis and sperm functions, as they are the key regulators of the cellular defense against OS [127,128,129]. In humans, functional polymorphisms in the antioxidant genes NRF2, SOD, GST, NOS, CAT, and GPX have been linked to male infertility [128].

Seminal OS has been linked to reduced sperm concentration, motility, and functions [130]. Studies show that cytokines, which are oxidative damage mediators, may also simultaneously affect the quality of sperm and male fertility. Roles of some cytokines in male fertility are reliant on their concentration; for example, Naz and Evans (1998) found that IL12 was connected to sperm density and morphology [131]. IL6 levels were shown to be higher in infertile males in a previous study by the same investigators. The presence of high amounts of specific cytokines in semen is frequently associated with a reduction in semen quality [132].

## 4. Oxidative Stress and Male Infertility

In about half of all instances, the cause of male infertility is unknown, but it is clear that 30–80% of infertile men have a significant concentration of ROS in their ejaculate. Because of the strong link between OS and male infertility, Agarwal et al. coined the term “Male Oxidative Stress Infertility (MOSI)” to describe male infertility caused by OS [133].

The two major sources of endogenous ROS in human semen are leukocytes in the seminal fluid and immature sperm with a morphologically deficient head and cytoplasmic retention [134,135,136]. During male genital tract infection, extrinsic ROS are produced, and leukocyte chemotaxis and activation promote additional inflammatory responses. To combat infections, leukocytes activate the myeloperoxidase system, which produces ROS [137]. OS in the seminal fluid can be caused by excessive ROS production by leukocytes. Abnormal and immature spermatozoa, on the other hand, are the generators of intrinsic ROS. Cytoplasm accumulates in the mid-piece during the normal spermiogenesis process, producing cell elongation and condensation. Excess residual body, which includes large amounts of cytosolic glucose-6-phosphate dehydrogenase (G6PD) enzyme and generates intracellular nicotinamide adenine dinucleotide phosphate, is retained by immature spermatozoa with morphological defects (NADPH). The intramembrane NADPH oxidase NOX5 then converts NADPH to ROS [138].

Disruption in the homeostatic balance between ROS and antioxidant defence systems can lead to the development of OS when highly reactive ROS outpace antioxidant defence mechanisms. It has been shown to have negative effects on sperm, including LPO, sperm DNA fragmentation (SDF), and germ cell apoptosis.

### 4.1. Lipid Peroxidation (LPO)

Spermatozoa have an abundance of polyunsaturated fatty acids (PUFAs), particularly docosahexaenoic acid, which has six double bonds in its non-conjugated methylene groups and is found in high concentrations in their plasma membrane. Because of the increased generation of ROS, PUFA peroxidation in sperm membranes is promoted, which results in cell dysfunction as a result of impaired membrane integrity and fluidity, which are required for effective fusion of sperm oocytes following capacitation and biochemical acrosome reaction cascade [15,16]. Additionally, LPO by-products harm the mitochondrial proteins that are involved in the electron transport chain, causing electron leakage and a decrease in mitochondrial membrane potential, ATP production, and sperm motility, among others [139,140]. The initial step of LPO is referred to as “initiation”, and it refers to the extraction of hydrogen atoms from carbon-carbon double bonds in an unsaturated fatty acid to create free radicals. The second phase is referred to as “propagation”, and it entails the formation of lipid radicals as well as their fast interaction with oxygen to create peroxyl radicals [141]. Because peroxyl radicals may snatch a hydrogen atom from an unsaturated fatty acid when metals such as copper and iron are present in the environment, a lipid radical and lipid hydrogen peroxide can occur when metals such as copper and iron are present [142]. The last step is referred to as “termination”, and it is during this phase that the radicals created react with subsequent lipids to produce harmful aldehydes and other end products. LPO produces number of main products, the most notable of which are malondialdehyde (MDA), 4-hydroxynonenal (4-HNE), and acrolein. MDA is a critical biomarker in the study and monitoring of PUFA peroxidation [143,144].

### 4.2. Sperm DNA Fragmentation

Increased ROS production and poor antioxidant capabilities in sperm might result in SDF [15]. OS has the ability to damage sperm DNA either directly or indirectly through the activation of sperm caspases and the production of endonuclease. It is believed that SDF is brought on by DNA susceptibility caused by a chromatin condensation mistake during the process of spermiogenesis, which results in the failure of the chromatin structure substitution from histone to protamine. It has been observed that excessive ROS exposure occurs during spermiation, during the migration of spermatozoa via the rete testis, from the seminiferous tubules to the cauda epididymis, and results in DNA damage [16,145]. The production of 8-OH-guanine and 8-OH-2′-deoxyguanosine (8-OHdG), an oxidised guanine adduct, is the consequence of this reaction. Increased amounts of 8-OHdG have been related to DNA fragmentation and strand breaks in the laboratory [121].

Single- and double-stranded (ds-) DNAs are both vulnerable to DNA fragmentation [15,140]. Throughout the spermiogenesis process, DNA repair can only take place at particular phases, and it is no longer active during the process of nuclear condensation of the epididymis. Despite the fact that the human oocyte represents the next chance for ss-DNA break repair, the effectiveness of SDF repair declines with increasing maternal age [146]. When DNA repair does not occur, a break in the ds-DNA leads to genome instability and subsequently induces cellular death [147]. A detrimental influence on embryo development and pregnancy outcome is thought to be caused by the presence of unrepaired SDF over a certain threshold, which is known as the “late paternal effect” [148]. At the 2nd day of human embryo development (4-cell stage), significant activation of embryonic genome expression occurs in a cleavage-stage embryo, and embryogenesis shifts from maternal factor dependency to embryo’s own genome dependence [149]. Consequently, after fertilisation, the presence of SDF in a spermatozoon can have a negative influence on blastulation, implantation, and pregnancy outcomes. The researchers also discovered that OS has a detrimental impact on the development of cleavage embryos, which they referred to as the “early paternal effect” [150]. Several studies have intervened into the impact of SDF on ART outcomes [151,152]. According to a meta-analysis, there is an inverse relationship between SDF and pregnancy outcome and a positive relationship between SDF and miscarriage [153]. Since SDF is one of the factors that contribute to recurrent pregnancy loss, proper measurement and management might help to alleviate the problem for couples.

### 4.3. Apoptosis

As a result of DNA fragmentation caused by a variety of cell death signalling and regulatory systems, apoptosis is recognised as a biologically programmed cell death [154]. Apoptosis may occur as a result of ds-DNA breaks induced by ROS. ROS also causes mitochondrial membrane disruption, resulting in the release of the cytochrome-C signalling molecule, which is capable of activating apoptotic caspases and annexin-V phosphatidylserine-binding activity [155]. Infertile men may suffer significant mitochondrial damage as a result of high levels of cytochrome-C present in their seminal plasma [156].

## 5. Oxidant Sensitive Inflammatory Pathways

Until the advent of male infertility research in the previous few decades, the mechanisms of inflammation-mediated male reproductive disturbances remained as disregarded concepts [157]. The study showed that microbial cell wall component and pathogenic factor (lipopolysaccharide, or LPS)-induced systemic inflammation which adversely affected male reproduction in rat model [157]. Following up on this discovery, numerous studies were conducted to investigate the possibility of inflammation-induced male infertility [6,8,12,13]. Increased production of pro-inflammatory mediators such as ROS, IL1 and TNF-α, NO, and prostaglandins reduce hypothalamic luteinising hormone (LH) secretion, pituitary luteinising hormone production, and Leydig cell cholesterol mobilisation, resulting in a reduction in steroidogenic functions. Additionally, inhibiting the steroidogenic activity of Leydig cells are inflammation-activated neurological pathways and corticosteroids, which are generated in response to inflammation and serve as anti-inflammatory agents [158,159]. However, reports on inflammation-mediated male infertility are not adequate and provide contradictory pieces of a larger jigsaw mechanism.

### 5.1. Initiation of Male Reproductive Tract Inflammation

Pathogen-associated molecular patterns (PAMPs), which are found on bacterial, viral, fungal, and protozoan pathogens, are recognised by the immune system and result in an inflammatory response during infection. This recognition is mediated by specific pattern recognition receptors (PRRs). For the most part, these receptors have been well-studied, and they have been shown to recognise nucleic acids from bacteria and viruses, as well as other pathogen-specific compounds including LPS, glycosaccharides, and bacterial lipopeptides [160,161]. Myeloid cells (macrophages, monocytes, and dendritic cells) are the primary source of TLR expression; however, they are also found in epithelial and connective tissue cells. Among the testicular cells, TLR expression is the greatest in the Sertoli cells. TLR ligand stimulation of Sertoli cells may activate inflammatory signalling pathways, such as the myeloid differentiation primary response (MYD88), that result in the activation of mitogen-activated protein kinases (MAP kinases) and the activation of inflammatory transcription factors, such as the nuclear factor of activated T cells (NFkB) and interferon regulatory factor 3 (IRF3) [162,163]. This results in the activation of gene expression patterns that are generally linked with inflammation, such as IL1α (the cell-associated form of IL1B), IL6, NO synthase 2 (NOS2 or inducible NOS, iNOS), as well as the immunoregulatory cytokine activin A [162,164,165]. The Sertoli cells have the ability to react to several of the endogenous inflammatory mediators, including TNF-α, type 1 and type 2 interferons, IL-1A and IL1B, NO, and transforming growth factor beta 3 (TGFB3) [166,167]. The evidence suggests that these molecules, which are commonly associated with inflammation, as well as the pathways through which they exert their effects, play critical roles in facilitating intercellular communication within the seminiferous epithelium [168,169]. This group of inflammatory mediators regulates spermatogenic cell mitosis and meiosis, as well as the development of the Sertoli cell cytoskeleton and intercellular connections, as well as a variety of Sertoli cell activities, during the whole cycle of the seminiferous epithelium. This causes spermatogenesis to be disrupted because pathogenic molecules, cytokines, and other inflammatory mediators produced inside the testis or entering the bloodstream resulting from systemic inflammation interfere with the normal activities of the Sertoli cells and spermatogenic cells, and as a result, spermatogenesis is disrupted. In addition, Leydig cells produce number of PRRs, which identify pathogenic components and activate inflammatory pathways in response to them. TLRs were the first identified receptors among these PRRs, and they were discovered in Leydig cells of the mouse for the first time. TLR3 and TLR4 are highly expressed in Leydig cells, and these cells have a long lifespan [170]. It is believed that innate immunity system of these cells, which is mediated by TLR3, is responsible for the activation of NF-κB and IRF3. This is followed by production of proinflammatory cytokines, including IL-6 and TN-α, as well as IFN-α and β. Activation of the TLR3 and TLR4 pathways is known to inhibit testosterone production in Leydig cells, which is mediated by the effect of TLR-induced high levels of cytokines, TNF-α and IL6, which are produced in the cells [171,172]. TLRs are found throughout the epithelium of the reproductive tract, including the epididymis, in addition to the testis, and are thought to be involved in the production of testosterone. However, data from several rat studies indicate that PRRs are expressed differently depending on their location, with TLRs 1–6 being expressed most strongly in the testis and TLRs 7, 9, and 11 being expressed more strongly in the epididymis, vas deferens, or both [162,163,164,165].

### 5.2. Inflammation-Mediated Oxidative Stress in Male Reproductive Tissues

**Figure 2:**
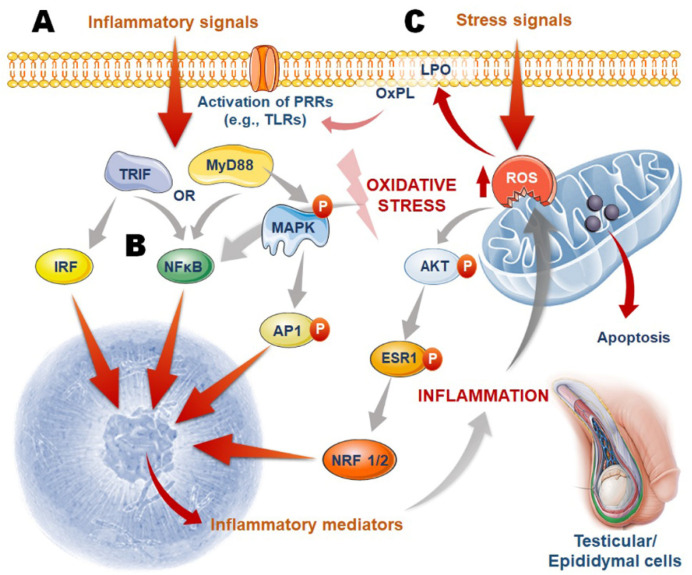
Cellular pathways connecting inflammation and oxidative stress (OS) in the pathogenesis of male infertility. (**A**) Pattern recognition receptors (PRRs) in testicular and epididymal cells are activated by inflammatory stimuli. The activated PRRs trigger downstream signalling via the Myeloid differentiation primary response (MYD88) and mitogen-activated protein (MAP) kinases pathways (IRF3); (**B**) These cascades activate transcription factors such as the nuclear factor kappa light chain enhancer of activated B cells (NF-kB), activator protein 1 (AP-1), and interferon regulatory factor 3 (IRF3); (**C**) Excess ROS production, on the other hand, can cause oxidation of membrane phospholipids and intracellular proteins, which can activate the PRRs-inflammatory pathway. Furthermore, ROS can activate the transcription factors nuclear respiratory factor (NRF) 1 and 2 via AKT (Protein Kinase B) and estrogen receptor (ESR) 1. These activated transcription factors induce the expression of inflammatory mediators such as tumour necrosis factor α (TNFα), interferons (IFNs), interleukin (IL) 1, nitric oxide (NO), and tumour growth factor (TGF) B3, which cause exaggerated inflammation and can also act as OS stimuli, creating a feedback loop. OS can also initiate apoptotic cascades by assisting the release of cytochrome-c (Cyt-c) from mitochondria.

In accordance with the findings mentioned above, TLRs predominantly activate the NF-κB pathway, which results in inflammatory responses and the development of OS (Figure 2). During inflammation, the testicular macrophages play a crucial role in the stimulation of inflammatory agents and ROS, which in turn disrupt gonadal steroidogenesis, cause testosterone levels to drop, and therefore interfere with normal spermatogenic activity [173]. If the testicles are in good health, testicular macrophages contribute to the testicular immune privilege while also exhibiting reduced inflammatory activity [174]. In men suffering from infertility-related orchitis or other inflammatory conditions, the number of macrophages with high phagocytic activity in testicular interstitial tissue is reduced, whereas the number of macrophages secreting copious amounts of inflammatory cytokines is increased in the testicular interstitial tissue. These macrophages respond to TLR-induced activation of p38 MAPK and NF-κB, which is followed by the release of inflammatory chemicals and the formation of OS, which results in testicular OS and death [175]. All of the effects of inflammation-induced oxidative damage to Leydig cells, including disruption of mitochondrial physiology, inability of the steroidogenic acute regulatory protein (StAR) to stimulate cholesterol transport into mitochondria, and suppression of steroid hormone synthesis, are caused by OS [176]. The generation of NO is also affected. It is known as guanosine triphosphate cyclohydrolase I (GCHI) because it is a rate-limiting enzyme in the synthesis of tetrahydrobiopterin, which is necessary for the activation of iNOS. The NF-κB pathways are also involved in the regulation of iNOS transcription [177]. NF-κB activity is controlled by the direct contact of TLR4 with NADPH oxidase 2 (Nox2), one of the NADPH oxidase subunits, which in turn is controlled by the direct interaction of TLR4 with NADPH oxidase 1 (Nox1), which in turn is controlled by the direct interaction of TLR4 with Nox1 [178].

### 5.3. Oxidative Stress-Induced Inflammation in Male Reproductive Tissues

In the same way that the inflammatory process may cause OS, the latter can also cause inflammation by activating number of different signalling pathways. Several studies have shown that the ROS, hydrogen peroxide can cause inflammation by activating the transcription factor NF-kB [31,32]. OS, in addition, has been shown to have a significant role in the activation of the NOD-like receptor protein 3 (NLRP3) inflammasome [179,180,181]. The NLRP3 inflammasome is an oligomeric molecular complex that activates innate immune responses by generating proinflammatory cytokines such as IL1 and IL18 [182]. Several methods of ROS-mediated activation of the NLRP3 inflammasome have been discovered lately. ROS produced by damaged mitochondria have been demonstrated to activate NLRP3 inflammasomes, resulting in the production of IL1 and the development of focal inflammation [179]. NLRP3 inflammasomes have also been reported to be activated during apoptosis when mitochondrial DNA has been oxidised [180]. Moreover, with induction of OS, ROS induces the thioredoxin-interacting protein, which inhibits endogenous antioxidant thioredoxin, thereby aiding its dissociation from thioredoxin and binding with the NLRP3 inflammasome, causing its activation.

It has also been demonstrated that DNA base alteration caused by ROS can cause inflammation. A signalling cascade is activated by the base excision repair of oxidatively damaged/modified DNA base (7,8-dihydro-8,8-guanine) by the 8-oxoguanine-DNA glyoxalase-1, which results in the activation of the NF-kB pathway, inducing expression of pro-inflammatory genes and the accumulation of inflammatory cells [183]. 

It has been discovered that the 8-isoprostane, an end product of arachidonic acid that belongs to the F2-isoprostanes family and is a marker of OS, can increase the expression of the pro-inflammatory chemokine IL-8 in human macrophages through the activation of MAP kinases. Moreover, it has been demonstrated that the extracellular redox potential of cysteine (Cys) and cysteine disulfide (CySS) induced by OS can be used to stimulate monocyte adhesion to vascular endothelial cells, activate NF-κB, and increase the expression of the proinflammatory cytokine IL-1 [184,185] (Figure 2).

Finally, OS-induced male infertility, as well as the mechanisms that underlie it, have been thoroughly studied. OS changes the parameters of sperm and has an impact on their functions and morphology through the processes of LPO of the sperm membrane, intracellular oxidative damage to spermatozoa, sperm DNA damage, and the triggering of apoptotic pathways in the germ cells [27].

## 6. Conclusions and Future Perspectives

The present study explains how inflammation and OS are interlinked in the pathogenesis of male infertility. Inflammatory stimuli activate PRRs in testicular and epididymal cells, leading to activation of transcription factors. Excess ROS production, on the other hand, can cause oxidation of membrane phospholipids and intracellular proteins, which can activate the PRRs-inflammatory pathway as well. The activated transcription factors boost the expression of inflammatory mediators which cause exaggerated inflammation and can also act as OS stimuli, creating a vicious feedback loop. However, more studies are needed to fill in certain gaps in concepts on inflammation and OS-induced male infertility pathogenesis. Future molecular research should emphasise on revealing the exact crosstalk among the testicular somatic and immune cells in maintaining the specialised innate immune system of the testes, how the systemic inflammation induces immune responses in the male reproductive tract. Moreover, with ageing increased, inflammation of the male urogenital tract is also known to account for infertility in men. Further, cytokines and adipokines in age-induced obesity adversely effects fertility in men. Thus, understanding the involvement of multiple immunoregulatory variables linked to ageing, as well as the influence of age-related changes on male reproductive health, is also critical. Studies are also encouraged in investigating various immunomodulators in mitigating the inflammation-induced OS causing male infertility/subfertility.
